# Does Eucalyptus Prevent Fever

**Published:** 1881-02

**Authors:** 


### DOES EUCALYPTUS PREVENT FEVER.

In the last number of *Nature* some very positive statements are made as to the value of the eucalyptus or blue gum tree of Tasmania in destroying fevers in marshy districts. The testimony in support of this power, it says, is most convincing. In marshy districts near eucalyptus forests fever seems to be unknown, and in parts of Corsica and Algeria, where the tree has been planted for the sake of its reputed virtues, endemic fevers have been stamped out. M. Gimbert, in a report to the French Academy, instanced the case of a farm situated in a pestilential district about 20 miles from Algiers, where by planting a number of trees the character of the atmosphere was entirely changed. Similar testimony comes from Holland, the south of France, Italy, California and many other parts of the world as to the febrifugal attributes of this tree.

In no case is the evidence more convincing than in that of Algeria, as related by Dr. Santra, and quite recently by Consul Playfair. Large tracts of land have been transformed by the agency of the "fever-destroying tree," as it has come to be called, and wherever it is cultivated fevers are found to decrease in frequency and intensity. Fewer districts in Europe have a more evil reputation than the Campagna as a veritable hot-bed of pestilential fever, and the people who know the country around Rome may remember the monastery at Tre Fontand, on the spot, as the tradition tells, that St. Paul met his death. Life in this monastery meant death to the monks; but, since the eucalyptus has been planted in the cloisters, fever has disappeared, and the place has become habitable.

---

To Keep Lamp Chimneys From Cracking.—The following recipe for keeping lamp chimneys from cracking is taken from the *Diamond*, a Leipzig journal devoted to the glass interest.: Place your tumblers, chimneys or vessels, which you desire to keep from cracking in a pot filled with cold water, add a little cooking salt, allow the mixture to boil well over a fire, and then cool slowly. Glass treated in this way is said not to crack even if exposed to very sudden changes of temperature. Chimneys are said to become very durable by this process, which may also be extended to crockery, stoneware, procelain, etc. The process is simply one of annealing, and the slower the process, especially the cooling portion of it, the more effective will be the work

### MOUNTAIN CLIMBING.

The greatest altitude which has been reached by mountain climbing was attained in Cashmere by Mr. Johnson, who some years ago mounted to a spot 22,300 feet above the sea. Aeronauts have ascended 30,600 feet and returned in safety. It is supposed by mountaineers that 25,000 to 26,000 feet is the utmost height that will ever be trod by human steps. That life can be supported at this altitude has been proved by the adventurers who have dared the dangers of the upper air in a balloon. During the last summer Mons. Werner ascended Mount Illimani, one of the loftiest peaks of the Bolivian Andes. The height of this mountain has been variously estimated, Mr. Pentland giving it an altitude of 24,200 feet ; Mr. Minchin setting it at 21,224 feet, and Mr. Werner himself making it to be only 20,812 feet. Few ascents to the height of 21,000 feet have been recorded. Hunters of the Himalayas often chase their game to the height of 20,000 feet, and natives living near Mount Demarend, near Teheran, frequently climb to the summit, about 20,000 feet, to gather sulphur from the crater.

---

Infection from Mosquitoes.—The discovery that mosquitoes carry filaria in their probosces, and infect the human subject with that much-dreaded worm parasite, has attracted considerable attention among the English microscopists. The matter has been brought before the Quekett Microscopical Club, by Dr. Cobbold, the President, who is one of the highest authorities on this subject. Particulars of various cases were given in which it was proved that those suffering from filaria had received the contagion from mosquitoes, and mosquitoes themselves infected with filaria were shown. Filaria are very minute worm-like parasites, which, on entering the human body, breed until they increase to countless numbers. By recent advices we learn that they have the power of entering and leaving the blood at pleasure ; they usually invade the circulation about seven o'clock in the evening, and increase until about midnight, after which time they retire to other parts of the system.

---

A gentleman called professionally on a prominent physician and complained of a total loss of appetite. "I'll just give you a little tonic to take before dinner," said the doctor. "Oh, I'm all right just before dinner. Its after dinner that I suffer so much."

### KITCHEN ECONOMY AGAIN.

LATER TESTS MADE BY THE GOVERNMENT CHEMIST.

The analytical chemist for the Indian Department of the government, Dr. Edward G. Love, has made further analyses of baking powders, and this time of samples both of which were purchased by Dr. Love himself in open market.

As carbonic acid gas is the bread leavening power generated by the admixture of cream of tartar and bi-carbonate of soda, the following, copied from Dr. Love's certificate of analysis of the comparative yield of this gas by the powders examined, is of interest:

| Name of the Baking Powder. | Available carbonic acid gas, cubic inches per each ounce of powder. |
|---|---|
| "Cleveland's Superior." | 118.2 |
| "Royal." | 116.2 |

The sample of Cleveland's Baking Powder previously analyzed, with result shown in the original article on "Kitchen Economy," was furnished to Dr. Love by the Royal Baking Powder Company.—*New York Tribune, Jan.* 28, 1881.

Dr. Gill and the Old Woman.—An old lady of his flock once called upon him with a grievance. The doctor's neck-bands were too long for her ideas of ministerial humility, and after a long harangue on the sin of pride, she intimated that she had brought her scissors with her, and would be pleased, if her dear pastor would allow her, to clip them down to her notions of propriety. The doctor not only listened patiently to her lecture, but handed her over the offending white bands for her to operate upon. When she had cut them to her satisfaction and returned the bibs, it was the doctor's turn.

"Now," said he, "my good sister, you must do me a good turn also." "Yes, that I will, doctor; what can it be?" "Well, you have something about you which is a deal too long, and causes me no end of trouble, and I should like to see it shorter." "Indeed, dear sir, I will not hesitate; what is it? Here are the scissors, use them as you please." "Come, then," said the sturdy divine, "good sister, put out your tongue."—*From the Life of Spurgeon.*

"That fellow is just like a telescope," said a dashing New York girl. "You can draw him out, see through him, and shut him up again."

### FOUR WOMEN.

The Boston *Traveller* tells a story of four sisters, who were left orphans by the death of their father, a Vermont farmer. They had a farm up in the mountains, which, at the best, yielded but a scanty income. Their only brother was a young boy, for whom they were ambitious. With a little money and a great fund of energy they came to Boston. Not to put their names on the books of some "Women's Union," or semi-charitable institution as "highly-educated young ladies," who want lady-like employment, and sit about for something to turn up, finding fault, meantime, with a cold, unfeeling world. They took a house in the business part of the city, took what boarders they could accommodate, but made the *piece de resistance* of their enterprise—day board.

They opened two parlors, which were for a dining-room. They are carpeted, draped, picture-hung, and made generally refined, and people like to come to them. The tables are arranged with the most scrupulous neatness. The linen is dainty and always fresh ; the silver is bright ; the details in every respect are those of a refined home. The meals are not elaborate, but everything is excellent of its kind, perfectly cooked and perfectly served.

With the aid of one servant, these four young women manage their establishment. In dainty, white ruffed aprons they serve the guests at their table in a graceful, ladylike way that attracts people. They have placed their young brother in a good school, they are making a comfortable support, and have their own pleasant home all together.

---

At Rochester, Mich., they have a good way of advertising the young men who stand on the church steps after meeting to stare at the ladies. The following card is kept standing in the *Era*, of that place : "The Donkey Club, of this city, would inform the young ladies especially, and the public generally, that they have made arrangements for an extensive demonstration on the steps in front of the Methodist Episcopal Chapel—the members locating themselves on either side of the main entrance—on Sunday evening next. Positions taken immediately after the close of the religious exercises within."

---

A Western dealer received a fresh invoice of mouse traps the other day, and advertised, "Grand arrival of spring goods."

